# Meta-analysis of the relationship between dietary starch intake and enteric methane emissions in cattle from *in vivo* experiments

**DOI:** 10.5455/javar.2024.k767

**Published:** 2024-03-31

**Authors:** Agustin Herliatika, Yeni Widiawati, Anuraga Jayanegara, Rakhmad Perkasa Harahap, Diana Andrianita Kusumaningrum, Mohammad Ikhsan Shiddieqy, Wahidin Teguh Sasongko, Sharli Asmairicen, Maureen Chrisye Hadiatry, Alif Shabira Putri, Eko Handiwirawan, Tatan Kostaman, Lisa Praharani, Umi Adiati

**Affiliations:** 1 Research Center for Animal Husbandry, Research Organization for Agriculture and Food, National Research and Innovation Agency, Cibinong Science Center, Bogor, Indonesia; 2 Department of Nutrition and Feed Technology, Faculty of Animal Science, IPB University, Bogor, Indonesia; 3 Study Program of Animal Science, University of Tanjung Pura, Pontianak, Borneo, Indonesia

**Keywords:** Cattle, enteric methane, *in vivo*, meta-analysis, starch

## Abstract

**Objective:**

Different sources and levels of starch in the total mixed ration might result in different rumen fermentation profiles, rumen microbial composition, and enteric methane production. The current study aimed to evaluate the effect of dietary starch intake on enteric methane production through a meta-analytical approach by integrating related published studies.

**Materials and Methods:**

Papers that provided study results on enteric methane production from cattle fed different sources and levels of starch were selected. A total of 52 publications were filtered based on some specified criteria, comprised of 73 studies focused on enteric methane production in cattle fed a basal diet supplemented with starch. The collected data were subjected to statistical meta-analysis through a mixed methodology model. The *p*-value and root mean square error (RMSE) were applied as the statistical models.

**Results:**

Results showed that increasing the level of dietary starch intake and its digestibility in the rumen as well as increasing propionate significantly decreased the acetate to propionate (A/P) ratio as well as enteric methane production (*p* < 0.01).

**Conclusions:**

It is concluded that the addition of starch to diets mitigates enteric methane production from cattle, primarily through two ways, i.e., manipulation of the protozoa population and alteration to a lower A/P ratio.

## Introduction

The FAO data report for 2020 shows that enteric fermentation emissions contribute 17.50% of total gas emissions in agrifood systems [1], and 71.67% of enteric fermentation emissions came from cattle [2]. Enteric fermentation emissions, as a product of methanogen activities in the rumen, have an unfavorable impact on the cattle and the environment. This is because the methane produced during methanogenesis in the rumen reflects the amount of dietary energy consumed by animal losses during the rumen microbial fermentation process [3]. The lower the methane produced during methanogenesis, the more dietary energy is used for animal production [4]. This lost energy will be released in the form of methane (CH_4_) into the environment, thereby worsening global warming.

Many approaches have been widely used to mitigate enteric methane production from ruminants and are classified into several groups. The first approach is rumen fermentation modification using various types of materials, such as bacteria [5,6], chemicals [7,8], oil [9], and plant active ingredients, namely tannins and saponins [10–14], that are added to animal feed as a feed additive. The second approach is high-quality feed sources for low-quality diet supplementation, such as high-protein or starch feed [15,16] and macroalgae supplementation [17–19]. The last approach is a nutrient balance in the complete ratio to ensure high feed efficiency that improves animal productivity [20]. Evaluations through meta-analysis of mitigation approaches using a modification of enteric methane production have been done previously [21,22].

One of the approaches used to mitigate enteric methane is starch supplementation in feed. Starch is commonly used as a source of energy in ruminant feed. Also, it improves the utilization of structural carbohydrates, which then increases the amount of protein flow to the small intestine [23]. Sources of starch mainly used in ruminant feeds are cereal grains, legumes, and tubers. Cereal grains such as corn, wheat, oats, and barley are primarily starch sources due to their high starch contents (60%–80%). Tubers like cassava contain around 60%–90% of starch, while legume beans contain 25%–50% of starch [24]. Grains as a source of feed energy have commonly become the main component in concentrate, improving fermentation kinetics, improving microbial protein supply flow into the small intestine, and decreasing methane formation in the rumen [25,26]. A high-starch diet in the rumen changes rumen bacteria composition by promoting propionic acid bacteria growth over methanogens [27]. Feeding ruminant animals with a high-starch diet depresses methanogen growth due to the lower rumen pH as a result of the rapid digestion process of starch diets [25,28].

A similar meta-analysis of starch used as feed in ruminants has been published previously by Moharrery et al. [29]. This paper discusses the relationship between starch intake used as feed for ruminants and its digestibility in the rumen, small intestine, and hindgut of dairy cows. Meanwhile, this study observes the relation of starch intake to feed digestibility, short-chain fatty acid profiles, rumen microorganism population, and enteric methane production. Furthermore, the present study aims to analyze the relationship between animal productivity and feed modification using starch to reduce enteric methane production through the meta-analysis method.

## Materials and Methods

### Ethical approval

This is a meta-analysis study, and no live animals were used in this study, so ethical approval is not necessary for this type of study.

### Search strategy

**Figure 1.**
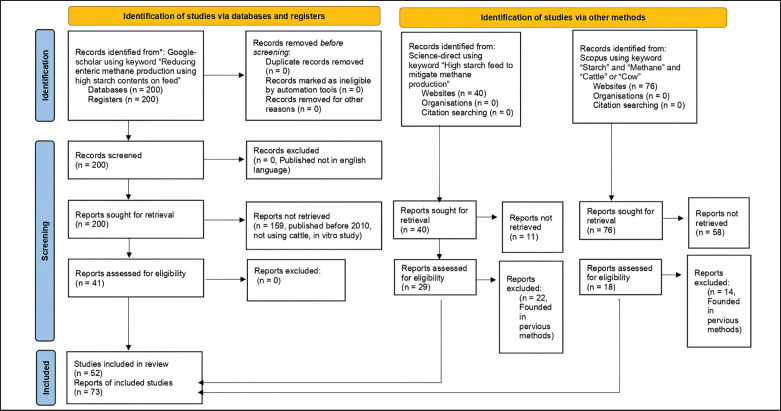
Process of paper selection according to PRISMA.

A database was developed from publishing papers mentioning the role of high starch content in diets to reduce enteric methane yield. A total of 302 publications were used in developing the database. The papers collected consisted of 200 publications found in Google Scholar using the keyword “Reducing enteric methane production using high starch contents on feed;” 40 publications found in Science Direct using the keyword “High starch feed to mitigate methane production,” and 76 publications found in Scopus using the keywords “starch” and “methane” and “cattle” or “cow.” This process was shown in the preferred reporting items for systematic reviews and meta-analysis (PRISMA) flow chart (Fig. 1).

### Selection criteria

All publications were selected based on the following criteria: 1) the article was published in English; 2) the year of publication was limited from 2010 to the newest; 3) the kinds of experiments were *in vivo*; 4) the animal used in the experiment was cattle; and 5) enteric methane production was not estimated using an equation but measured directly using the green feed, respiratory chamber, and SF6 systems. Some experiments measuring the effect of feeding starch on enteric methane emissions were eliminated from the list of publications collected since the animals used in the experiment were sheep, goats, and buffalo.

### Database development

A total of 52 publications were selected based on the above criteria, consisting of 73 studies that focused on the cattle chosen (Fig. 1). When the experiment consisted of more than one study, each respective study was encoded separately. The number of cattle used in each experiment reported in those publications started from three heads and up to 28 heads, with a mean of 7,8 cows in each experiment. Variables used and integrated into the database developed were methane production, dry matter (DM) intake, starch intake, volatile fatty acid (VFA) total and partial production, bacteria, and protozoa population, DM, neutral detergent fiber (NDF), and acid detergent fiber (ADF) digestibility. The dietary starch as the main parameter used in the analyses was selected based on the type of sources. They are mixed grain or concentrate, corn meal or grain as a single feedstuff, wheat, barley, rapeseed meal, and soybean meal.

The level of starch supplement calculated in the database was expressed in grams per kg of DM. When the amount of starch given to the animals in the experiments was expressed in different units, they were then calculated to make the same unit (gm/kg DM). The level of starch supplemented with the daily rations was variable, ranging from 5 to 603.23 gm/kg DM. The population of bacteria and protozoa variables were logarithmized to allow a linear relationship with dependent variables. The units of bacteria and protozoa population were expressed as log_10_ cells/gm DM rumen content and log_10_ cells/ml rumen fluid.

There was also variation in the enteric methane production measurement period among the different systems. The methane production was measured from one to seven days into the period when the respiratory chamber was used. Measurements were conducted over a period of 4–112 days when greenfeed units were used. When the SF6 method was used in the experiment, the measurements were taken for 4–15 days. Once we evaluated all the selected publications to meet the specified criteria, we listed the results in Table 1.

### Statistical analysis

The data collected in the database was processed using statistical meta-analysis based on mixed model methodology. Different studies were classified as random effects; the intake of starch was classified as a fixed effect. This study uses a linear mixed model with a statistical model based on a *p*-value (<0.05). All statistical analyses were carried out using SAS University Edition. The following model was applied [82,83]:

*Y_ij_ = B_0_ + S_i_ + B_1_X_ij_ + b_i_X_ij_ + e_ij_*(1)

where:

*Y_ij_* = Dependent variable expected on study *i* and level *j* of *X* (independent variable),

*B_0_* = The overall intercept across all studies (fixed effect),

*S_i_* = The random effect of study *i*th (*i* = 1, …, 73),

*B_1_X_ij_* = The overall regression coefficient of *Y* on *X_ij_* (*X* on *i* study and *j* level), also called slope,

*b_i_X_ij_* = The random effect of *i* study on the regression coefficient of *Y* on *X_ij_*,

*e_ij_* = The unexplained residual error.

Data were analyzed to express: 1) the effect of feed-starch intake and metabolic BW on enteric methane production (gm/kg DMI) and short-chain fatty acids; 2) the correlation among feed digestibility, enteric methane production (gm/kg DMI), and rumen microorganism; and 3) the effect of VFA concentration on enteric methane production (gm/kg DMI) and rumen microorganism. The model statistics presented are the *p-*value and root mean square error (RMSE). The data were then reported following PRISMA guidelines [84].

## Results

### The effect of feed-starch intake and metabolic BW on enteric methane production and short-chain fatty acids

The linear regression generated based on data collected from in vivo studies between feed-starch and methane production is presented in Table 2. The feed starch was expressed as total intake (gm/head/day), intake per body weight (BW) gain (gm/BW^0.75^), and starch contained in the feed (gm/kg DM intake). The results indicated that there is a positive correlation between metabolism, BW, and enteric methane production. Increasing metabolic BW significantly increased enteric methane produced (*p* < 0.05). Meanwhile, results showed a negative correlation between starch intake, both as a total and per metabolic BW, and enteric methane production. Increasing starch content in the diet and starch intake significantly decreased enteric methane production (*p* < 0.01). When starch intake was expressed per metabolic BW, there was a considerable decrease in enteric methane production. The enteric methane production was decreased by 0.05343 gm/kg DMI for every 1 gm starch intake per BW^0.75^.

**Table 1.** *In vivo* experiments (Expt.) included in this meta-analysis.

Study	Authors	Starch source	Basal feed	Level of starch (gm/kg DM)	Animal	Gas sampling (Methods)	Gas sampling period (Days)
1	[30]	Grain mix (concentrate)	Forage:Concentrate (55:45 w/w), corn silage versus grass silage, and grain mix versus pulp mix	22–382	Lactating Holstein cows	SF6 tracer technique	6
2	[30]	Grain mix (concentrate)	Forage:Concentrate (55:45 w/w), corn silage versus grass silage, and grain mix versus pulp mix (with and without *Propionibacterium* addition)	22–382	Lactating Holstein cows	SF6 tracer technique	6
3	[30]	Grain mix (concentrate)	Forage:Concentrate (55:45 w/w), corn silage versus grass silage, and grain mix versus pulp mix (with and without *Propionibacterium+Lactobacillus plantarum* addition)	22–382	Multiparous Lactating Holstein cows	SF6 tracer technique	6
4	[30]	Grain mix (concentrate)	Forage:Concentrate (55:45 w/w), corn silage versus grass silage, and grain mix versus pulp mix (with and without *Propionibacterium+Lactobacillus rhamnosusu* addition)	22–382	Multiparous Lactating Holstein cows	SF6 tracer technique	6
5	[31]*	Wheat (concentrate)	Forage:Concentrate (50:50 w/w), fiber-rich concentrate versus starch-rich concentrate	56.52–218.75	Multiparous Lactating Holstein cows	Respiration chamber	6
6	[31]*	Wheat (concentrate)	Forage: Concentrate (50:50 w/w), Fiber-rich concentrate versus starch-rich concentrate (with and without bicarbonate 1%DMI addition)	55.56–227.68	Multiparous Lactating Holstein cows	Respiration chamber	6
7	[32]*	Corn meal	Forage: Concentrate (51.7:48.3 w/w), corn meal versus soybean hulls	238.00–280.00	Lactating second porous Italian Friesian cows	Respiration chamber	1
8	[32]*	Corn meal	Forage: Concentrate (51.7:48.3 w/w), corn meal versus soybean hulls (with and without fish Oil addition)	232.00–274.00	Lactating second porous Italian Friesian cows	Respiration chamber	1
9	[33]	Corn silage	Forage: Concentrate (80:20 w/w), grass silage versus 33%, 67%, and 100% of corn silage, respectively, as forage	5.00–262.00	Multiparous lactating Holstein-Friesian cows	Respiration chamber	5
10	[34]	Corn grain (slowly fermentable) and Gelatinized corn grain (rapidly fermentable)	Forage: Concentrate (60:40 w/w), Concentrate contains 27% and 53% either slowly fermentable starch or rapidly fermentable starch, respectively.	110.00–217.00	Multiparous lactating Holstein-Friesian cows	Respiration chamber	5
11	[35]	Corn	Forage:concentrate (79.84:20.16 w/w) for feed contains corn in the concentrate and (79.73:20.27 w/w) for feed contains soybean hulls	163.00–352.00	Nellore bulls	SF6 tracer technique	4
12	[35]	Corn	Forage: concentrate (81.56:18.44 w/w) for feed contains corn in the concentrate and (81.4:18.6 w/w) for feed contains soybean hulls, both with oil (ether extract) from soybean meal	110.00–479.00	Nellore bulls	SF6 tracer technique	4
13	[36]*	Corn silage	Forage:concentrate (45%:55% w/w) or or (35%:65% w/w), rice straw versus corn silage	202.61–262.41	Multiparous Chinese Holstein dairy cows	Respiration chamber	2
14	[37]	Wheat/NaOH-treated wheat	Forage:concentrate (49.4%:50.6% w/w), wheat versus sugar beet molasses and (49%:51% w/w), NaOH treated wheat versus sugar beet molasses+Sodium bicarbonate	12.00–249.00	Lactating Danish Holstein dairy cows	Respiration chamber	4
15	[38]*	Corn grain or wheat grain	Forage: concentrate (53.49: 46.51 w/w), with a concentrate containing 8 kg of corn grain or wheat grain, respectively (with and without 0.8 kg of canola oil 0.8 kg supplementation)	213.09–237.12	Lactating, multiparous, rumen-cannulated Holstein-Friesian cows	SF6 tracer technique	7
16	[39]	Barley and rapeseed cake	Forage:concentrate (68:32 w/w) and (39:61 w/w)	105.00–218.00	High refusal feed intake (RFI) and low RFI Holstein dairy	Respiration chamber	2
17	[39]	Barley and rapeseed cake	Forage:concentrate (68:32 w/w) and (39:61 w/w)	105.00-218.00	High RFI and low RFI Jersey Dairy Cows	Respiration chamber	2
18	[40]	Refined starch	Forage: concentrate (61:39 w/w), 10% refined starch versus dextrose, both contain 11%RDP in ration	193.00–281.00	Multiparous Holstein dairy cows	Greenfeed	4
19	[40]	Refined starch	Forage: concentrate (61:39 w/w), 10% refined starch versus dextrose, both contain 9%RDP in ration	193.00–282.00	Multiparous Holstein dairy cows	Greenfeed	4
20	[41]	Whole crop-wheat silage (WCW), concentrate (contains Rolled barley)	Forage: concentrate (75.4:24.6 w/w), (77.2:22.8 w/w), (77.15:22.75 w/w), (76.3:23.7 w/w), (71.6:28.4 w/w), and (12.1:87.9 w/w) for treatments WCW with different ratio of grain to straw plus chaff (11:89, 21:79, 31:69, and 47:53), grass silage, and ad libitum concentrate grass silage	71–444	Continental crossbred steers (predominantly Charolais and Limousin	SF6 tracer technique	5
21	[42]*	Rapeseed meal, maize grain, barley grain, oat grain, extruded mixture (50% linseed, 30% wheat bran, and 20% sunflower meal), soybean meal	Forage:Concentrate (22.4:77.6 w/w) versus (0:100 w/w) during the first 200 days	69.28–297.22	Charolais bulls	SF6 tracer technique	15
22	[42]*	Rapeseed meal, maize grain, barley grain, oat grain, extruded mixture (50% linseed, 30% wheat bran, and 20% sunflower meal), soybean meal	Forage:Concentrate (22.4:77.6 w/w) versus (0:100 w/w) during 24 days of feedlot	69.28–297.22	Charolais bulls	SF6 tracer technique	15
23	[42]*	Rapeseed meal, maize grain, barley grain, oat grain, extruded mixture (50% linseed, 30% wheat bran, and 20% sunflower meal), soybean meal	Forage:Concentrate (22.4:77.6 w/w) versus (0:100 w/w) during 120 days of feedlot	69.28–297.22	Charolais bulls	SF6 tracer technique	15
24	[42]*	Rapeseed meal, maize grain, barley grain, oat grain, extruded mixture (50% linseed, 30% wheat bran, and 20% sunflower meal), soybean meal	Forage:Concentrate (22.4:77.6 w/w) versus (0:100 w/w) during 228 days of feedlot	69.28–297.22	Charolais bulls	SF6 tracer technique	15
25	[43]*	Whole rapeseed/whole cottonseed/whole linseed	Forage: Concentrate (63:37 w/w), (61:39 w/w), (50:50 w/w), and (61:39 w/w) for prilled fatty acids, whole rapeseed, whole cottonseed, and whole linseed, respectively.	197.21–211.39	Multiparous Holstein Friesian cows	SF6 tracer technique	6
26	[44]*	Crushed corn grain/crushed wheat/single-rolled barley/double-rolled barley	Forage: concentrate (53:47 w/w), concentrate contains one of the grains (single-rolled corn, single-rolled wheat, single-rolled Barley, or double-rolled Barley)	240.27–313.51	Lactating multiparous Holstein-Friesian cows	SF6 tracer technique	5
27	[45]*	Crushed wheat	Forage: Concentrate (88.95:11.05 w/w), (75.12:24.88 w/w), (60.1:39.9 w/w), and (44.22:55.78 w/w) for a concentrate that contains 0, 3, 6, and 9 kg of wheat, respectively	34.74–331.16	Lactating, multiparous Holstein-Friesian cows	SF6 tracer technique	3–5
28	[46]*	Grounded corn grain/corn silage+soybean meal	Forage:Concentrate (60:40 w/w), contained 0%DM, 28.2%DM, dan 56.4%DM corn silage	169.59–300.88	Multiparous lactating Holstein cows fitted with ruminal cannulas	Respiration chamber	3
29	[47]*	Corn silage+corn grain+soybean meal/corn grain+soybean meal	Forage: Concentrate (49:51 w/w), (63:37 w/w), and (14:86 w/w), for ration contains natural grassland hay, corn silage, and wheat straw as forage, respectively.	290.80–480.83	Young Blond d‘Aquitaine Bulls,	SF6tracer technique	5
30	[48]*	Maize silage	Forage: Concentrate (65:35 w/w), concentrate control with one of forage. The forage is early grass silage, late grass silage, and maize silage.	43.18–144.89	Lactating Danish Holstein dairy cows	Respiration chamber	4
31	[48]*	Maize silage	Forage: Concentrate (62.6:37.4 w/w), high fat concentrate with one of forage. The forage is early grass silage, late grass silage, and maize silage.	43.93–138.10	Lactating Danish Holstein dairy cows	Respiration chamber	4
32	[49]*	Corn silage	Forage: Concentrate (80:20 w/w), of which 70% is grass silage (leafy, boot, early heading, and late heading)	76.43–82.76	Lactating Holstein-Friesian dairy cows (low intake)	Respiration chamber	3
33	[49]*	Corn silage	Forage: Concentrate (80:20 w/w), of which 70% is grass silage (leafy, boot, early heading, and late heading)	77.38–82.84	Lactating Holstein-Friesian dairy cows (high intake)	Respiration chamber	3
34	[50]*	Barley grain (steamrolled)+canola meal/corn-based dried distillers grains with solubles (CDDGS)/wheat-based dried distillers grains with solubles (WDDGS)	Forage: Concentrate (55:45 w/w), which concentrate contains 40% barley gain+canola meal, CDDGS, WDDGS, and WDDGS+corn oil, respectively	174–358	Spayed crossbreed beef heifers	Respiration chamber	4
35	[51]	Barley grain (steamrolled), grounded barley grain, linseed ground	Forage: concentrate (47.5:52.5 w/w), use grass hay as forage either with or without 15% grounded linseed supplementation	244–314	Non-pregnant non-lactating and ruminally cannulated Holstein cows	SF6 tracer technique	3
36	[51]	Barley grain (steamrolled), grounded barley grain, barley silage, linseed ground	Forage: concentrate (47.5:52.5 w/w), use barley silage as forage either with or without 15% grounded linseed supplementation	314–363	Non-pregnant non-lactating and ruminally cannulated Holstein cows	SF6 tracer technique	3
37	[52]	Maize silage+soybean meal	Forage:concentrate (69.5:30.5 w/w), (64.8:35.2 w/w), and (69.5:30.5 w/w) for control, supplementation 4%DM of linseed oil, and 2.71%DM of calcinit (Nitrate source), respectively	208–223	13 Multiparous lactating Holstein and 6 Holstein-Jersey cross dairy cows	Respiration chamber	2
38	[52]	Maize silage+soybean meal	Forage: concentrate (60:40 w/w), for all treatments (control, supplementation 4%DM of linseed oil, and 2.71%DM of calcinit as Nitrate source)	167–180	Rumen-cannulated Holstein-Friesian lactating dairy cows	Respiration chamber	2
39	[53]*	Corn grain, wheat bran, linseed meal, with or without extruded linseed: wheat bran (70:30)	Forage:concentrate (50:50 w/w), use hay as main forage with 0%DM, 5%DM, 10%DM, and 15%DM of extruded linseed inclusion	93.47–112.98	Lactating multiparous Holstein cows	SF6 tracer technique	4
40	[53]*	Corn grain, wheat bran, linseed meal, with or without extruded linseed: wheat bran (70:30)	Forage:concentrate (50:50 w/w), use corn silage as main forage with 0%DM, 5%DM, 10%DM, and 15%DM of extruded linseed inclusion	228.74–271.12	Lactating multiparous Holstein cows	SF6 tracer technique	4
41	[54]*	Dry rolled barley grain/CDDGS/WDDGS	Forage: concentrate (8:92 w/w), 41) which concentrate contains dry rolled barley grain or substitution 40%DM of dry rolled barley grain using CDDGS/WDDGS/WDDGS and corn oil	319–550	Crossbreed beef heifers	Respiration chamber	7
42	[55]	Corn silage, ground corn grain, with or without solvent soybean meal and expeller soybean meal	Forage: concentrate (55:45 w/w), which forage is Alfalfa silage and Corn Silage with proportions 20:80; 40:60; 60:40; and 80:20, respectively	240–296	Multiparous lactating cows	Respiration chamber	3
43	[56]*	Barley silage+rolled barley grain+soybean meal/barley silage+corn silage+rolled barley grain+griund corn grain+corn gluten meal+soybean meal/corn silage+griund corn grain+corn gluten meal+soybean meal	Forage: concentrate (54.4:45.6 w/w), in which corn silage replace the barley silage in level 0%DM, 27.2%DM, and 54.4%DM	169.41–258.78	Multiparous lactating Holstein cows fitted with rumen cannulas	Respiration chamber	3
44	[57]*	Flaked corn, soybean meal, with or without DDGS	Forage:concentrate (60.1:39.9 w/w), with 0%DM, 10%DM; 20%DM, and 30%DM supplementation of DDGS	112.30–184.19	Multiparous lactating Holstein cows fitted with rumen cannulas	Respiration chamber	3
45	[58]	Concentrate (oats, barley, peas, rapeseed cake, wheat bran, rapeseed)	Forage:Concentrate (50:50 w/w), (70:30 w/w), and (90:10 w/w)	35–173	Rumen-fistulated swedish red breed (SRB) dairy cow	SF6 tracer technique	5
46	[59]	Corn silage. Cron grain, soybean meal expeller, with or without soybean meal solvent	Forage: concentrate (53.6:46.4 w/w), (55.3:44.7 w/w), (66.9:33.1 w/w), and (69.1:30.9 w/w) for low forage which contains NDF 19% and high forage which contains 24%, for both proportion Alfalfa silage: Corn silage 70:30 and 30:70, respectively	221–230	12 Primiparous Holstein (606 kg) and 12 Primiparous Jersey (407 kg)	Greenfeed	4
47	[60]	Fresh alfalfa/fresh sainfoin	Forage: concentrate (100:0 w/w), for Fresh Alfalfa and Fresh Sainfoin in level 20:80 and 0:100 when both harvested in the early stage and late stage, respectively	9–14	Beef Heifers	Respiration chamber	3
48	[60]	Fresh alfalfa/fresh sainfoin	Forage: concentrate (100:0 w/w), for Alfalfa Hay or Sainfoin Hay	7–21	Beef Heifers	Respiration chamber	3
49	[61]*	Corn silage	Forage: concentrate (80:20 w/w), the feed contains 75% corn silage which is harvested at a DM content of 25%, 28% corn, 32%, and 40%	243.33–326.67	Holstein-Friesian dairy cows (8 primiparous and 20 multiparous)	Respiration chamber	5
50	[62]*	Corn silage+ground corn grain+soybean meal/corn silage	Forage: concentrate (57:43 w/w), forage is Red Clover silage or corn silage, both with and without 4% DM of linseed oil supplementation	103.43–248.32	Multiparous lactating Holstein cows fitted with rumen cannulas	Respiration chamber	3
51	[63]	Maize silage/ryegrass silage/ or both silages	Forage: concentrate (63:37 w/w), (69:31 w/w), and (63:37 w/w) for feed contains both low starch and fat, high starch and low fat, and both high starch and fat, respectively	180–252	Multiparous lactating Holstein cows	Greenfeed	112
52	[64]	Wheat, corn	Forage: concentrate (50:50 w/w), for control, inclusions of 3% calcium nitrate, inclusions of 4% linseed oil, and inclusions of 3% calcium nitrate+4% linseed oil, respectively	253–257	Multiparous non-lactating Holstein cows	Respiration chamber	4
53	[65]	Wheat, corn	Forage: concentrate (50:50 w/w), for control, inclusions of 0.5% tea saponin, inclusions of 2.3%nitrate, and Inclusions of 0.5% tea saponin +2.3% nitrate, respectively	254–264	Multiparous non-lactating Holstein cows	Respiration chamber	4
54	[66]	Extruded linseed	Forage: concentrate (60:40 w/w), for control and inclusions of 9.8% extruded linseed + 2.4% calcium ammonium nitrate	280–288	Lactating Holstein cows	Respiration chamber	2
55	[67]*	Steam flaked corn (SFC) with or without wet distillers grains with solubles (WDGS)	Forage: concentrate (10:90 w/w), concentrate contains SFC and substitute of SFC using WDGS at levels 0%DM, 15%DM, 30%DM, and 45%DM, respectively	386.38–603.23	Jersey steers	Respiration chamber	5
56	[68]	Corn silage/grain sorghum silage with corn meal, soybean meal, and concentrate mix (21% canola meal, 21% cotton seeds, 19% barley meal, 8% soybean flakes)	Forage: concentrate (56.4:43.6 w/w), (51.6:48.4 w/w), and (42.9:57.1 w/w) for forage that contains corn silage, substitute corn silage using whole plant grain sorghum silage or forage sorghum silage, respectively	250–268	Cows	Respiration chamber	7
57	[69]	Maize silage	Forage: concentrate is (71.84:28.16 w/w), (71.51:28.49 w/w), and (68.81:31.19 w/w) for feed containing Maize silage LG30224 (low starch, high NDFd), Maize silage falkone (high starch, low NDFd), and Maize silage falkone (high starch, low NDFd)+Maize meal, respectively	204–226	Lactating Frisian Holstein Cows	Respiration chamber	4
58	[70]	Rolled barley, ground wheat	Forage: concentrate (65:35 w/w) or (35:65 w/w), both with and without 5%DM of Sunflower oil	110–318	Multiparous Nordic red cows	SF6 tracer technique	6
59	[71]*	Soybean meal with or without maize silage, cracked wheat	Forage: concentrate (75:25 w/w), forage is maize silage or grass silage, both with and without 6%DM supplementation of extruded linseed	105.20–357.37	Holstein-Friesian dairy heifer	Respiration chamber	4
60	[71]*	Soybean meal with or without maize silage, cracked wheat	Forage: concentrate (60:40 w/w), forage is maize silage or grass silage, both with and without 5%DM supplementation of extruded linseed	163.07–263.16	Holstein-Friesian dairy heifer	Respiration chamber	4
61	[72]*	Soybean meal with or without ground corn grain	Forage: concentrate (54.4:45.6 w/w), forage is Alfalfa silage which substitutes with Timothy silage at level 0%DM, 27.2%DM, and 54.4%DM	136.89–186.59	Multiparous lactating Holstein Cows	Respiration chamber	5
62	[73]	Maize grain/gelatinized maize	Forage: concentrate (60:40 w/w), concentrate contains 37%DM or 53%DM of slowly fermentable starch and rapidly fermentable starch	110–217	Multiparous lactating Holstein-Friesian cows	Respiration chamber	5
63	[74]*	Maize silage. High starch concentrate (contains barley, wheat feed, sunflower meal, maize gluten meal) or low starch concentrate (contains maize distillers grains and maize gluten meal)	Forage: concentrate (71.36:28.64 w/w), for two kinds of concentrate (concentrate-rich fiber and concentrate-rich starch), both with different ratios of Grass silage and Maize Silage (30:70 and 70:30)	98.55–194.22	Multiparous lactating Holstein-Friesian cows	SF6 tracer technique	5
64	[75]	Barley, rapeseed meal, wheat starch	Forage:concentrate (60.5:39.5 w/w), feed contains 20%DM of wheat starch or 20%DM refined Glycerol	–	Swedish red dairy cows	Greenfeed	7
65	[76]	Maize silage, cracked wheat, wheat feed, soybean meal, rapeseed meal, with or without maize meal	Forage: concentrate (50:50 w/w), forage contains different levels of grass silage and maize silage (75:25 or 25:75 w/w), both with and without inclusion of 5%DM of extruded linseed	186–223	Multiparous lactating Holstein-Friesian cows	Respiration chamber	4
66	[77]	Ground corn	Forage: concentrate (60.87:39.13 w/w), (60.16:39.84 w/w), (61.67:38.33 w/w), and (61.11:38.89 w/w) for feed contains ground corn in the concentrate or for feed contains soybean hulls, both with and without oil (ether extract) from soybean meal, respectively	32.9–172	Nellore bulls	SF6 tracer technique	6
67	[78]	Maize silage, concentrate (contains 22.2% maize, 20% hominy feed, 12.5% maize gluten feed, 10% formaldehyde-treated soybean meal, and 6.2% soybean meal)	Forage:concentrate (52.5:47.5 w/w) and (52.2:47.8 w/w) for control and Inclusion mixture of linseed oil (1.5%DM)+lauric acid (0.4%DM)+myristic acid (1.2%DM)+Ca fumarate (0.7%DM), respectively	165–168	Lactating Holstein-Frisian dairy cows	Respiration chamber	7
68	[79]	Wheat, corn, with or without soybean meal, formaldehyde-treated soybean meal	Forage:concentrate (66:34 w/w) for concentrate contains 3.5%DM of Urea or 8.8%DM of Nitrate source	234–236	Lactating Holstein-Frisian dairy cows	Respiration chamber	7
69	[80]	Corn silage, grain mix (contains 14.1% barley, 10.9% wheat, and 9.2% corn)	Forage: concentrate (55:45 w/w), forage is corn silage+hay or grass silage	18–274	Lactating primiparous Holstein cows	Respiration chamber	2
70	[80]	Corn silage, grain mix (contains 14.1% barley, 10.9% wheat, and 9.2% corn)	Forage:concentrate (55:45 w/w), forage is corn silage+hay or grass silage, both supplemented with Propionibacterium freudenreichii 53 W	18–274	Lactating primiparous Holstein cows	Respiration chamber	2
71	[80]	Corn silage, grain mix (contains 14.1% barley, 10.9% wheat, and 9.2% corn)	Forage: concentrate (55:45 w/w), forage is corn silage+hay or grass silage, both supplemented with Lactobacillus pentosus D31	18–274	Lactating primiparous Holstein cows	Respiration chamber	2
72	[80]	Corn silage, grain mix (contains 14.1% barley, 10.9% wheat, and 9.2% corn)	Forage: concentrate (55:45 w/w), forage is corn silage+hay or grass silage, both supplemented with Lactobacillus bulgaricus D1	18–274	Lactating primiparous Holstein cows	Respiration chamber	2
73	[81]	Barley silage rolled corn grain	Forage:concentrate (55:45 w/w), feed contains 0%DM, 1%DM, 2%DM, and 3%DM of encapsulated Nitrate	392–401	Rumminaly cannulated beef heifer	Respiration chamber	3

w/w = Weight/weight, SF6 = Sulfur hexafluoride, RFI = Refusal feed intake, RDP = Rumen-degradable protein, WCW = whole crop-wheat silage, DMI = dry matter intake, DM = dry matter, CDDGS = corn-based dried distillers grains with solubles, WDDGS = wheat-based dried distillers grains with solubles, DDGS = dried distillers grains with solubles, SFC = steam flaked corn, WDGS = wet distillers grains with solubles, NDFd = neutral detergent fiber digestibility, *level of starch (gm/kg DM) was calculated from data in the literature.

**Table 2.** The effect of feed-starch intake and metabolic BW on enteric methane production (gm/kg DMI) in cattle.

Independent variable	Unit	*n*	Parameter estimates			Model statistics		
			Intercept	SE intercept	Slope	SE slope	*p*-value	RMSE
Intake starch	gm/head/day	222	22.3	0.613	−0.00065	0.00013	<0.0001	6.17
Intake starch	gm/BW^0.75^	207	21.4	0.616	−0.053	0.016	<0.0001	6.37
Starch contained in feed	gm/kg DMI	224	21.8	0.595	−0.0079	0.0019	<0.0001	6.31
BW^0.75^	kg	219	16.7	3.16	0.029	0.026	<0.0001	6.40

*n =* number of samples, SE = standard error, RMSE = root mean square error, BW = body weight, BW^0.7^ = metabolic body weight, kg = kilogram, DMI = dry matter intake.

**Table 3.** The effect of feed-starch intake on short-chain fatty acid profiles in the rumen.

Independent variable	Unit	n	Parameter estimates			Model statistics		
			Intercept	SE intercept	Slope	SE slope	*p*-value	RMSE
Starch intake (gm/head/day)								
Acetate	%	157	67.2	0.809	−0.0011	0.00016	<0.0001	6.50
Propionate	%	153	18.3	3.90	0.0015	0.00037	<0.0001	13.8
Butyrate	%	157	12.8	0.423	−0.00014	0.000083	<0.0001	3.30
A/P		157	3.93	0.130	−0.022	0.0031	<0.0001	1.07
Starch Intake (gm/BW^0.75^)								
Acetate	%	153	66.8	0.775	−0.132	0.019	<0.0001	6.52
Propionate	%	153	19.2	3.93	0.173	0.045	<0.0001	14.1
Butyrate	%	153	12.8	0.405	−0.0188	0.0099	<0.0001	3.34
A/P		161	4.01	0.135	−0.00019	0.000026	<0.0001	1.06
Starch Content in Feed (gm/kg DMI)								
Acetate	%	157	67.6	0.732	−0.021	0.0024	<0.0001	6.08
Propionate	%	157	18.4	3.78	0.026	0.0056	<0.0001	13.7
Butyrate	%	157	12.4	0.407	−0.00037	0.0013	<0.0001	3.36
A/P		161	3.96	0.127	−0.0030	0.00040	<0.0001	1.06

*n =* Number of samples, SE = Standard error, RMSE = Root mean square error, A/P = Acetate/Propionate, BW = Body weight, BW^0.7^ = Metabolic body weight

The results of linear regression to indicate the correlation between starch contained in the diet and starch intake with VFA concentration are presented in Table 3. Increasing starch content in the diet and consumption of starch, both total and per metabolic BW, significantly increased the concentration of propionate (*p* < 0.01). The opposite results were found on the correlation between starch intake and concentrations of acetate and butyrate, as well as a ratio of acetate to propionate (A/P). Increasing starch content in the diet and starch intake significantly decreased the A/P ratio (*p* < 0.01).

### The correlation among feed digestibility, enteric methane production (gm/kg DMI), and rumen microorganisms

Analyzed results on the effect of DM, OM, NDF, and ADF digestibility on enteric methane production are presented in Table 4. There was a positive correlation between the digestibility of DM, OM, NDF, and ADF and enteric methane production. Increasing digestibility of DM, organic matter (OM) NDF, and ADF significantly increases the methane production in the rumen (*p* < 0.01). A different result was shown on the correlation between starch digestibility and methane production in the rumen. There was a negative correlation between starch digestibility and enteric methane production. When the amount of starch digested in the rumen increases, the amount of methane produced in the rumen tends to decrease.

The result of analyses on the correlation between rumen microbial population and feed digestibility is shown in Table 5. Rumen bacteria and methanogen populations have a positive correlation with DM digestibility but have a negative correlation with OM, NDF, and ADF digestibility. However, based on the data collected and being meta-analyzed, the increase in the total population of rumen bacteria and methanogen did not significantly increase DM digestibility (*p* > 0.05). Moreover, those increases also did not significantly decrease NDF and ADF digestibility (*p* > 0.05) but decreased OM digestibility (*p* < 0.05). A different pattern was found for the correlation between protozoa population and digestibility. Increasing the rumen protozoa population significantly increased ADF and NDF digestibility (*p* < 0.05), while significantly decreasing DM, OM, and starch digestibility (*p* > 0.05).

Table 6 shows the results of the linear correlation between the rumen microbial population and the production of methane in the rumen. When rumen bacteria and methanogen were expressed based on DM content in the rumen, there was a negative correlation indicated between enteric methane production and the population of rumen bacteria and methanogen. Data showed that a decrease in enteric methane production occurred when the population of bacteria and methanogen bacteria increased (*p* < 0.05). A different result was identified in the correlation between the protozoa population and the methane produced in the rumen. When the population of protozoa was expressed based on ml of rumen fluid, increasing the population significantly increased the amount of methane produced in the rumen (*p* < 0.01).

### The effect of VFA concentration on enteric methane production and rumen microorganisms

The linear correlation between VFA concentration and enteric methane production is presented in Table 7. An increase in total VFA production caused a rise in enteric methane production (*p* < 0.05). However, when it was expressed per VFA individual, such as acetate, propionate, butyrate, minor VFA, and A/P, the results showed different patterns. Increasing the concentration of acetate, butyrate, minor VFA, and ratio A/P raised enteric methane production significantly (*p* < 0.01); except for acetate, the increase was not significantly different (*p* > 0.05). A negative correlation was shown for propionate concentration. Increasing propionate concentration resulted in a significant decline in enteric methane production (*p* < 0.01).

The results presented in Table 8 indicated a negative correlation between the four variables, namely the concentration of total VFA, acetate, butyrate, A/P ratio, and the rumen microbial population. On the other hand, the opposite results were found in the correlation between the propionate concentration, the minor VFA concentration, and the rumen microbial population. Increasing concentrations of total VFA, acetate, and butyrate tend to decrease the population of rumen bacteria and methanogen but tend to increase the population of rumen protozoa. However, the increase in acetate did not strongly increase the rumen protozoa population (*p* > 0.05). On the other hand, when the analyses were based on propionate, the increase in propionate concentration significantly increased the rumen bacteria and methanogen population but decreased the rumen protozoa population (*p* < 0.05).

## Discussion

Regardless of the different sources of starch used and the variation in the level of starch offered to the cattle, the results show that dietary starch content on the basal diet and increasing starch intake by cattle increased propionate production but decreased production of acetate, butyrate, and the A/P ratio in the rumen (Table 3). The results also show that increasing starch intake reduced the formation of methane in the rumen (Table 2). Those findings were coherent with those reported by Bannink et al. [85] and Hristov et al. [86] that enteric methane production in the rumen is influenced by dietary factors. Daily intake in the form of starch produces more propionate during the fermentation process in the rumen. Increasing propionate production decreases the H available for methane production in the rumen. It is the reason enteric methane production decreases when using higher starch feed. A study by Hatew et al. [34] also suggested that increasing the intake of starch mitigates the amount of methane formed in the rumen.

Results of the current study also indicated that an increase in propionate concentration was followed by a decline in acetate, butyrate, and the A/P ratio and a decline in the amount of methane produced in the rumen (Tabel 7). This is because, during the formation of acetate in the rumen, H_2_ was released and then became available in the rumen. Meanwhile, an amount of H_2_ is required during the formation of propionate in the rumen. For each molecule of acetate formed, two H_2_ molecules were also released. While four H_2_ molecules were required to produce one molecule of propionate [20,87]. This indicates that an increase in the amount of propionate formed will be followed by a decrease in the amount of acetate formed. On the other hand, methanogenic archaea are other microbiota that live in the rumen and use an amount of H_2_ to form enteric methane gas [88,89].

**Table 4.** The effect of nutrient digestibility on enteric methane production (gm/kg DMI).

Independent variable	Unit	*n*	Parameter estimates			Model statistics		
			Intercept	SE intercept	Slope	SE slope	*p*-value	RMSE
DM digestibility	%	135	13.8	5.89	0.082	0.085	0.0236	6.52
OM digestibility	%	158	16.2	5.32	0.048	0.074	0.0038	6.02
NDF digestibility	%	176	14.0	1.61	0.103	0.028	<0.0001	5.98
ADF digestibility	%	84	15.6	2.08	0.092	0.041	<0.0001	6.49
Starch digestibility	%	112	22.4	2.63	−0.026	0.027	<0.0001	5.92

DMI = dry matter intake, kg = kilogram, BW = body weight, *n =* number of samples, SE = standard error, RMSE = root mean square error, DM = dry matter, OM = organic matter, NDF = neutral detergent fiber, ADF = acid detergent fiber.

**Table 5.** The effect of rumen microorganism population on nutrient digestibility.

Independent variable	Unit	*n*	Parameter estimates			Model statistics		
			Intercept	SE intercept	Slope	SE slope	*p*-value	RMSE
Bacteria (log_10_ cells/gm DM rumen content)								
DM Digestibility	%	8	11.0	24.0	4.67	1.93	0.6774	2.23
OM Digestibility	%	8	96.7	16.1	-1.93	1.29	0.0093	1.13
NDF Digestibility	%	8	165	65.9	-9.53	5.29	0.0877	5.31
ADF Digestibility	%	8	183	77.8	-10.8	6.25	0.1004	6.69
Methanogen (log_10_ cells/gm DM rumen content)								
DM Digestibility	%	8	18.2	28.8	4.96	2.80	0.5729	2.27
OM Digestibility	%	8	98.5	17.7	-2.51	1.71	0.0114	1.22
NDF Digestibility	%	8	175	68.0	-12.5	6.59	0.0818	5.92
ADF Digestibility	%	8	188	82.5	-13.5	8.01	0.1075	7.60
Protozoa (log_10_ cells/ml rumen fluid)								
DM Digestibility	%	55	70.7	5.77	-0.362	1.07	<0.0001	5.99
OM Digestibility	%	55	71.9	5.74	-0.109	1.06	<0.0001	6.25
NDF Digestibility	%	57	26.6	11.1	4.87	2.05	0.0274	11.2
ADF Digestibility	%	47	39.9	16.5	1.89	3.06	0.0292	27.6

DMI = dry matter intake, *n =* number of samples, SE = standard error, RMSE = root mean square error, DM = dry matter, OM = organic matter, NDF = neutral detergent fiber, ADF = acid detergent fiber.

**Table 6.** The effect of rumen microorganism population on enteric methane production (gm/kg DMI).

Independent variable	Unit	*n*	Parameter estimates			Model statistics		
			Intercept	SE intercept	Slope	SE slope	*p*-value	RMSE
Bacteria	(log_10_ cells/gm DM rumen content)	14	105	18.3	−7.38	1.52	0.0022	5.76
Methanogen	(log_10_ cells/gm DM rumen content)	14	56.9	11.5	−4.19	1.19	0.0042	6.60
Protozoa	(log_10_ cells/ml rumen fluid)	75	7.53	2.74	2.05	0.463	0.0115	5.39

kg = kilogram, DMI = dry matter intake, *n =* number of samples, SE = standard error, RMSE = root mean square error, DM = dry matter

**Table 7.** The effect of VFA profile on enteric methane production (gm/kg DMI) in cattle.

Independent variable	Unit	*n*	Parameter estimates			Model statistics		
			Intercept	SE intercept	Slope	SE slope	*p*-value	RMSE
Total VFA	mM	145	20.0	2.02	0.0039	0.018	<0.0001	7.00
Acetate	%	157	3.77	4.24	0.265	0.067	0.3784	6.53
Propionate	%	157	20.8	0.639	−0.0099	0.018	<0.0001	6.46
Butyrate	%	157	15.6	1.86	0.408	0.146	<0.0001	6.67
Minor VFA	%	116	19.9	1.46	0.079	0.352	<0.0001	6.74
A/P		161	12.8	1.31	2.29	0.371	<0.0001	6.28

VFA = volatile fatty acids, kg = kilogram, DMI = dry matter intake, *n =* number of samples, SE = standard error, RMSE = root mean square error, mM, millimolar; A/P = acetate/propionate.

**Table 8.** The effect of VFA concentration on rumen microorganism population.

Independent variable	Unit	n	Parameter estimates			Model statistics		
			Intercept	SE intercept	Slope	SE slope	*p*-value	RMSE
Total VFA (mM)								
Bacteria	(log_10_ cells/gm DM rumen content)	14	13.3	0.734	−0.011	0.0060	<0.0001	0.339
Methanogen	(log_10_ cells/gm DM rumen content)	14	10.6	0.862	−0.0077	0.0067	<0.0001	2.02
Protozoa	(log_10_ cells/ml rumen fluid)	75	5.02	0.668	0.0064	0.0056	<0.0001	0.959
Acetate (%)								
Bacteria	(log_10_ cells/gm DM rumen content)	14	14.0	0.747	−0.032	0.012	<0.0001	0.281
Methanogen	(log_10_ cells/gm DM rumen content)	14	11.5	0.855	−0.029	0.012	<0.0001	0.302
Protozoa	(log_10_ cells/ml rumen fluid)	71	0.571	0.924	0.081	0.014	0.5430	0.725
Propionate (%)								
Bacteria	(log_10_ cells/gm DM rumen content)	14	11.5	0.261	0.029	0.0068	<0.0001	0.226
Methanogen	(log_10_ cells/gm DM rumen content)	14	9.17	0.409	0.025	0.0083	<0.0001	0.277
Protozoa	(log_10_ cells/ml rumen fluid)	71	7.64	0.346	−0.093	0.0092	<0.0001	0.556
ADF digestibility	(%)	47	39.9	16.5	1.89	3.06	0.0292	27.6
Butyrate (%)								
Bacteria	(log_10_ cells/gm DM rumen content)	14	13.2	0.230	−0.089	0.012	<0.0001	0.149
Methanogen	(log_10_ cells/gm DM rumen content)	14	10.6	0.433	−0.073	0.022	<0.0001	0.267
Protozoa	(log_10_ cells/ml rumen fluid)	71	3.24	0.397	0.212	0.026	<0.0001	0.657
Minor VFA (%)								
Bacteria	(log_10_ cells/gm DM rumen content)	8	10.3	0.207	0.488	0.046	<0.0001	0.080
Methanogen	(log_10_ cells/gm DM rumen content)	8	8.60	0.228	0.385	0.051	<0.0001	0.108
Protozoa	(log_10_ cells/ml rumen fluid)	54	6.04	0.339	−0.170	0.083	<0.0001	1.04
A/P								
Bacteria	(log_10_ cells/gm DM rumen content)	14	12.6	0.274	−0.165	0.048	<0.0001	0.257
Methanogen	(log10 cells/gm DM rumen content)	14	10.2	0.404	−0.161	0.047	<0.0001	0.253
Protozoa	(log_10_ cells/ml rumen fluid)	75	4.02	0.369	0.523	0.069	<0.0001	0.656

VFA = volatile fatty acids, *n =* number of samples, SE = standard error, RMSE = root mean square error, mM = millimolar; DM = dry matter, A/P = acetate/propionate.

In line with the above results, methane produced in the rumen increased due to more DM, OM, NDF, and ADF digested in the rumen, as presented in the digestibility data shown in Table 4. Different results were demonstrated for a correlation between the amount of starch digested in the rumen and the methane produced. Increasing the amount of starch digested leads to a reduction in methane formed by Methanogen archaea. Since the digestion of starch in the rumen produces more propionate [90], less H_2_ is available in the rumen. The proper availability of H_2_ is required for methanogenesis by methanogen archaea [91].

Increasing the intake of starch or starch contents in the diet leads to an increase in propionate, which increases the bacteria and methanogen archaea but decreases the total protozoa in the rumen. The decrease in protozoa population when the starch contents in the diet increased was also caused by the high passage rate of feed in the rumen. It was noted that dietary fiber was used as a place to hide for protozoa, which were multiplying very slowly. Therefore, if fiber content is restricted in feed, then the number of washed-out protozoa becomes high [88]. Some methanogens live in the rumen through association with protozoa; the range of association between protozoa and methanogen is about 0%–100% [92], so it is still possible that the number of methanogens increases and protozoa decreases with the inclusion of starch in the feed. Accordingly, protozoa provide hydrogen as a substrate for methanogenesis conducted by the methanogen archaea [93]. Thus, a reduction in the protozoa population may lead to a decline in the population of methanogen archaea and, subsequently, reduce methane emissions as well. The fermentation end products that are produced by protozoa are acetate, butyrate, and H_2_. The H_2_ is then used for methanogenesis by methanogen archaea to produce methane in the rumen [88]. When the number of protozoa decreases, there is limited H_2_ available in the rumen, resulting in a decrease in the amount of methane produced by methanogen [94]. Meanwhile, the number of bacteria is increasing, caused by decreasing the number of protozoa that engulf bacteria besides the feed particles [88].

The correlation between protozoa and NDF digestibility was explained by Puniya et al. [88]. Protozoa use the slow rate of passage of dietary fiber in the rumen as a place to hideout. Therefore, NDF digestibility will increase when the number of protozoa increases.

However, some datasets showed that starch inclusion in the feed does not affect enteric methane production. This is identified as a reason why the level of starch is not the only factor reducing enteric methane production. There may be different digestibilities of starch that can affect enteric methane production. Moharrery et al. [29] reported that total starch digestibility depended on the starch sources but did not affect starch intake. This study uses feed with a total starch digestibility higher than 90% (95.54% of the dataset), which results in increasing starch levels and decreasing enteric methane production. Therefore, a deeper observation of the starch digestibility effect on enteric methane production is needed, particularly for foods that have a total starch digestibility of less than 90% or come from different starch sources.

It should also be noted that what is included in the present meta-analysis study is starch, which comes from various sources. The degradation of starch in the rumen varied based on the type of starch and other nutrients contained in the feedstuff [95]. This means that other nutrients present in the diet might have influenced the degradability of starch in the rumen. Sutaryono et al. [96] reported that the inclusion of *Leucaena* in corn stover silage affected DMD and OMD in the *in vitro* study. This is due to the fact that cellulose is more resistant to digestion compared to starches [97]. Despite this, cellulose [98] and trace minerals [99,100] also affect feed digestibility. Similarly, the presence of other nutrients may also have different interactions with starch, resulting in variations in rumen microbial composition, VFA production, and methane generated in the rumen. This means that the presence of other nutrients cannot be neglected regarding their roles in the different results of the study presented.

## Conclusion

This current meta-analysis study demonstrated that, based on many experiments with different sources and levels of starch supplemented to the basal diets of cattle, the addition of starch in the diets mitigates methane produced in the rumen. There are two ways dietary starch mitigates enteric methane production: first, through manipulation of the protozoa population or defaunation, and second, through changing the proportion of acetate and propionate as expressed as a low A/P ratio in the rumen. The two mechanisms decrease H available in the rumen, which is required for methanogenesis by methanogen archaea.

## List of Abbreviations

A/P, acetate/propionate; ADF, acid detergent fiber; BW, body weight; CDDGS, corn-based dried distillers grains with solubles; DDGS, dried distillers grains with solubles; DM, dry matter; DMI, dry matter intake; H_2_, hydrogen; kg, kilogram; mM, millimolar; *n*, number of samples; NDF, neutral detergent fiber; NDFd, neutral detergent fiber digestibility; OM, organic matter; PRISMA, preferred reporting items for systematic reviews and meta-analysis; RDP, Rumen-degradable protein; RFI, refusal feed intake; RMSE, root mean square error; SE, standard error; SFC, steam flaked corn; SF6, sulfur hexafluoride; VFA, volatile fatty acid; w/w, weight/weight; WCW, whole crop-wheat silage; WDDGS, wheat-based dried distillers grains with solubles; WDGS, wet destiller grains with solubles.
